# Connecting Cholesterol Efflux Factors to Lung Cancer Biology and Therapeutics

**DOI:** 10.3390/ijms22137209

**Published:** 2021-07-05

**Authors:** Maria Maslyanko, Ryan D. Harris, David Mu

**Affiliations:** Leroy T. Canoles Jr. Cancer Research Center, Department of Microbiology and Molecular Cell Biology, Eastern Virginia Medical School, Norfolk, VA 23501, USA; maslyam@evms.edu (M.M.); harrisrd@evms.edu (R.D.H.)

**Keywords:** lung cancer, cholesterol efflux, ABCA1, ABCG1, Apo AI, miRNA, miR-33a, miR-200b-3p, LRPs, LAL, NPC1, STARD3, SMPD1, NCEH1, SR-BI, TTF-1, drug resistance, cisplatin

## Abstract

Cholesterol is a foundational molecule of biology. There is a long-standing interest in understanding how cholesterol metabolism is intertwined with cancer biology. In this review, we focus on the known connections between lung cancer and molecules mediating cholesterol efflux. A major take-home lesson is that the roles of many cholesterol efflux factors remain underexplored. It is our hope that this article would motivate others to investigate how cholesterol efflux factors contribute to lung cancer biology.

## 1. Introduction

Cholesterol is essential for cell viability and cell membrane integrity. Cholesterol is also a precursor to many physiologically important hormones. The interest in the interconnection between cholesterol metabolism and cancer is best illustrated by the abundance of literature on this subject matter. A quick search of PubMed.gov (accessed on 4 June 2021) using the two key words (Cholesterol AND Cancer) retrieves over 20,000 publications. Homeostasis of cholesterol within a single somatic cell is generally balanced by three major mechanisms: (i) de novo biosynthesis via the mevalonate pathway, (ii) import from extracellular sources, and (iii) efflux to remove cholesterol in excess. With regard to effluxes, there are four ways by which cholesterol could be effluxed from the cell: (i) passive diffusion, (ii) SR-B1-faciliated diffusion, (iii) active efflux by ABCA1, and (iv) ABCG1-mediated efflux [1]. The multiplicity of the cholesterol efflux mechanisms reflects the well-documented cytotoxicity of excess free cholesterol [2]. In this article, we choose to review the literature on this niche subject—the connections of known cholesterol efflux factors (Table 1) to lung cancer biology and therapeutics. Since microRNAs are clearly involved in the regulation of cholesterol efflux [3], they are included in this review paper as well. It is our hope that future research will be devoted to shedding more light on the fundamental roles of cholesterol efflux in lung cancer and the functional nuances of cholesterol efflux in a cancer type-dependent manner.

## 2. Genes Connected to Lung Cancer Biology by Multiple Lines of Evidence

### 2.1. ATP-Binding Cassette Transporter A1 (ABCA1)

Reverse cholesterol transport (RCT) is a complex process that results in the net movement of cholesterol from peripheral tissues back to the liver [4]. Cellular cholesterol efflux is mediated by high-density lipoprotein (HDL), acting in conjunction with lecithin, which acts as cholesterol acyltransferase [4]. Overexpression of major HDL components have been shown to be anti-atherogenic [4]. Additionally, increased levels of intracellular cholesterol create an environment conducive to tumor progression [5]. Upregulation and downregulation of various components of RCT pathway were observed in various cancer cell lines, impacting development of cancer, as well as treatment options [5,6,7,8,9,10,11]. Thorough investigation into the molecular mechanism of such changes is necessary for optimization of cancer treatment and improvement of the overall understanding of pathophysiology of cancer.

ATP-binding cassette transporter A1 (ABCA1) plays an essential role in RCT, acting as a phospholipid translocase, and contributing to the formation of a non-raft membrane domain that facilitates the lipidation of Apo AI and the formation of nascent HDL particles [12]. It is encoded by the *ABCA1* gene on human chromosome 9 (9q31.1) [13]. The gene was well described in various publications, as well as its mutations and associated medical conditions [14,15,16,17]. Under physiological conditions, *ABCA1* is predominantly regulated by oxysterols via the liver X receptor (LxR) pathway [18] and cyclic adenosine monophosphate (cAMP) [19].

Additional *ABCA1* regulatory mechanisms were as well described in various tissues [12]. In lung cancer cells, *ABCA1* is a direct transcriptional target of Thyroid transcription factor1 (TTF-1), which plays a crucial role in driving lung maturation and morphogenesis [20]. Since TTF-1 positively upregulated miR-33a [21], which is known to repress *ABCA1*, it is reasonable to expect inhibition of cholesterol efflux and elevation of the intracellular cholesterol levels with higher TTF-1 levels. However, upregulation of TTF-1 lowered intracellular cholesterol levels. This surprising finding was attributed to that fact that TTF-1 directly transactivates *ABCA1* [22]. The study of Lai et al. went on to show that TTF-1-high lung cancer cells of a lower intracellular cholesterol level are more sensitive to statins, raising the thesis that TTF-1 should be further investigated as a biomarker indicative of lung cancer vulnerability to statins. Midkine (MK), a low molecular weight protein and a product of a retinoic acid, is considered an important positive factor in inflammation, oxidative stress, and lipid metabolism. MK has been shown to inhibit cholesterol efflux from macrophages by reducing ABCA1 transporter expression, which suggests that ABCA1 may mediate multiple pathophysiological processes attributed to MK [23].

Research conducted over the past several years revealed additional connections between the ABCA1 transporter and lung cancer biology. Investigation of the therapeutic effect of efatutazone (an oral peroxisome proliferator-activated receptors (PPAR) agonist) using lung adenocarcinoma cells showed that efatutazone treatment increased mRNA and protein expression of PPAR-gamma, LxR-alpha and ABCA1, suggesting that efatutazone functions through the PPAR-gamma/LxR-alpha/ABCA1 pathway and that PPAR-gamma-LxR alpha pathway could mediate the expression of ABCA1 transporter [24].

Mechanism of ABCA1 involvement in the pathophysiology of cancer is complex. On the one hand, ABCA1 is a known tumor suppressor [25]. Loss of ABCA1 expression results in high intracellular cholesterol levels, which creates an environment conducive to tumor progression [5]. ABCA1 transporter is directly suppressed by miR-200b-3p [6]. Upregulation of miR-200b-3p expression was observed in lung adenocarcinoma. Therefore, it is proposed that one of the mechanisms of cancer cell proliferation and metastasis in lung tissues is via miR-200-3p-directed inhibition of the ABCA1 transporter [6].

On the other hand, ABCA1 has been reported to potentiate breast cancer metastasis by increasing membrane fluidity [7]. In one experiment, ABCA1 was shown to be overexpressed in 41% of metastatic tumors, reducing time to metastasis by 9 years [7]. The team demonstrated that increased membrane fluidity was a necessary feature of metastatic potential, and it was suggested that pharmacological control of the fluidity could potentially improve prognosis [7]. Additionally, overexpression of ABCA1 is associated with resistance to several medications in breast and lung cancer, including curcumin [8], doxorubicin [26], nitidine [27], and more. The proposed nature of the drug resistance is rapid transport of the chemotherapy agents out of the cells by ABCA1 transporter [8]. In one study, upregulation of RASSF1C in tumor cells downregulates miR-33a, consequently upregulating ABCA1 [28]. This desensitizes breast and lung cancer cells to the apoptotic effects of betulinic acid [29].

Another study investigated whether miR-106a was able to mediate resistance of the NSCLC cell line A549 to cisplatin (DDP) [30]. Reverse transcription quantitative polymerase chain reaction showed upregulation of miR-106a in the DDP-resistant cell line, and significantly reduced ABCA1 expression, suggesting that miR-106a directly targets *ABCA1* [30]. ABCA1 knockdown in the DDP-resistant cell lines that were overexpressing miR-106a significantly decreased the cancer cells’ apoptotic rate in the presence of DDP. This was a surprising finding, since it was previously believed that drug resistance was meditated by overexpressed ABCA1 via rapid transport of the chemotherapy agents out of the cells [8]. However, this experiment clearly showed downregulation of ABCA1 in DDP-resistant cell lines, suggesting that the role of ABCA1 in drug resistance is much more complex than what had been previously believed.

Several approaches may overcome the drug resistance dependent on the overexpression of ABCA1. Treatment with valproic acid (VPA) enhanced cisplatin sensitivity of non-small-cell lung cancer (NSCLC) cells via HDAC2 mediated down-regulation of ABCA1, further reinforcing the idea that overexpression of ABCA1 in lung cancer can be associated with resistance to therapies [31]. Another approach was used to overcome alpha-Tocopheryl succinate (alpha-TOS) resistance in NSCLC A549 cell line [32]. Resistance was overcome by using a mitochondrially targeted analogue of alpha-TOS, MitoVES, which is taken up based on the membrane potential, therefore bypassing the ABCA1 transporter altogether [32].

### 2.2. Apolipoprotein AI (Apo AI)

HDL plays crucial anti-oxidative, anti-inflammatory, anti-apoptotic and vasoprotective roles in various tissues [33]. Apolipoprotein AI (Apo AI) is a major component within HDL, accounting for up to 70% of its mass [12]. The interaction between Apo AI and ABCA1 is a rate-limiting first step of the RCT [12]. The exact nature of the interaction between Apo AI and ABCA1 transporter was well described in previous publications, as well as various mutations affecting Apo AI structure [12,34,35,36,37]. Recent advances have been made in discovering the exact structure of Apo AI and its intermolecular interactions in HDL [38]. The tertiary structure of Apo AI and its influence on Apo AI functionality in RCT pathway was further investigated [39]. Research showed that oxidative modification of Apo AI could also be a contributing factor in altering RCT levels [40,41]. Additionally, chlorination, but not nitration, of Apo AI through the myeloperoxidase pathway impaired the ability of Apo AI to interact with ABCA1 and to activate the Janus-kinase-2 signaling pathway, preventing Apo AI from promoting cellular cholesterol efflux in macrophages [42].

Overall, there is an inverse association between the base line HDL-cholesterol level and rate of cancer incidence [43]. For every 10-mg/dL increment in HDL-cholesterol, there was a 36% relatively lower rate of cancer incidence [44]. Another research team demonstrated that Apo AI and its mimetics effectively inhibited tumor development in a mouse model of ovarian cancer. They further reported that the mimetic peptide inhibited tumor angiogenesis by suppressing vascular endothelial growth factor (VEGF) and basic fibroblast growth factor (FGF) signaling pathways [45]. Inhibition of tumor development by Apo AI mimetics was also achieved in mouse models of colon cancer [46], B16F10L murine malignance melanoma model, and several other models [47]. The research team proposed that tumor growth suppression was due to the ability of Apo AI to induce infiltration of CD11b+ F4/80+ macrophages with an M1 anti-tumor phenotype, and also to decrease the number of myeloid-derived suppressor cells (MDSCs) [46].

A decreased level of Apo AI prior to the beginning of the cancer treatment was associated with a worse prognosis in patients with small cell lung cancer (SCLC) [9] and NSCLC [10]. A proposal was made to measure serum Apo AI before initial treatment as a biomarker to evaluate for metastasis and predict prognosis for SCLC [9] and NSCLC [10]. A correlation of Apo AI turnover with survival and response to first line platinum-based chemotherapy in advanced NSCLC was confirmed, and normalization of Apo AI was associated with a low risk of progression of cancer [48]. Another study showed that treating the intestine with the oral Apo AI mimetic Tg6F reduces tumor burden in mouse models of metastatic lung cancer [43].

### 2.3. ATP Binding Cassette Transporter G1 (ABCG1)

The ATP binding cassette transporter G1 (ABCG1) promotes the lipidation of HDL particles in a variety of tissues, playing a crucial role in the RCT pathway [12]. Research shows that ABCG1 increases in the cholesterol-loading conditions (following the treatment with acetylated low-density lipoproteins (LDL)), and decreases following treatment with HDL-3, confirming that it is regulated by the cellular cholesterol content [12]. The presence of a miR-33a binding site in the ABCG1 messenger RNA allows it to target *ABCG1*, marginally affecting its transcription [49,50]. ABCG1 is likely regulated by LxR pathway and the peroxisome proliferator-activated receptors (PPARs) [51]. Specifically, ABCG1 expression was reduced following overexpression of group X secretory phospholipase A2 [51].

ABCG1 plays a crucial role in homeostasis of the lung: deficiency of Abcg1 in mice results in accumulation of surfactants, lamellar body-loaded T2 cells, lipid-loaded macrophages, B-1 lymphocytes, and immunoglobulins [52].

Investigation into the high expression of the homeodomain transcription factor HOXB13 in chemoresistant lung adenocarcinoma showed an association with poor prognosis and resistance to cisplatin therapy [53]. Analysis revealed that HOXB13 upregulated a number of genes that are responsible for metastasis and drug-resistance, including *ABCG1, EXXH2* and Slug [53]. One of the possible mechanisms of the drug resistance involving ABCG1 transporter is rapid transport of chemotherapy agents out of the cells, similarly to ABCA1 transporter [8].

ABCG1 promoted proliferation of HKULC4 lung cancer cells by regulating their proliferation, apoptosis, and cancer stem cell-associated markers [11]. ABCG1 downregulated miR-29a, miR-29b and miR-29c expression in HKULC4 cells and promoted migration and invasion of cancer cells [11]. Another team showed lung cancer inhibition by betulinic acid nanoparticles via ABCG1 downregulation in HKULC2 cells [54]. Betulinic acid nanoparticles promoted expression of p21 and p53 and downregulated CD133, ALDH, BCL2, MCL1, ABCG1 and c-Myc expression, reducing cancer proliferation by approximately 33% [54]. Furthermore, genetic variations of *ABCG1* are associated with survival of NSCLC [55]. Therefore, ABCG1 should be further evaluated as a potential therapeutic target in NSCLC.

### 2.4. MicroRNAs (miRNAs)

MicroRNA (miRNA) are short RNA strands (approximately 22 nucleotides) that function as key regulators of lipoprotein metabolism within the cells [12]. MiRNAs regulate gene expression by targeting specific sequences within the 3′UTR of genes [12]. Several key miRNAs have been identified as regulators of RCT pathway, including miR-33, miR-106b, miR-7585 and miR-200b-3p [6].

Due to downregulation of miRNA-33a-5p in lung cancer cell, it was hypothesized that it functions as a tumor suppressor in lung cancer cells [28]. MiRNA-33a is known to impact expression of ABCA1 and ABCG1 transporters, regulating RCT within the cells [28]. RASSF1C was shown to downregulate miR-33a in lung cancer cells, therefore derepressing ABCA1 and ABCG1 transporters [28]. Such derepression of ABCA1 and ABCG1 may explain RASSF1C-dependent drug resistance since overexpression of ABCA1 and ABCG1 transporters is expected to enhance transport of the chemotherapy agents out of the cells [8,26,27,31]. Based on the observation that miR-33a is positively upregulated by TTF-1 [21], we suggest that TTF-1 may play a crucial role in the regulation of RCT pathway within the lung cells [22]. Mice carrying global miR-33 knockout are predisposed to obesity and insulin resistance. However, the role of SREBP-1 in mediating these phenotypes is controversial [56,57]. Interestingly, liver-specific ablation of miR-33 does not lead to increased body weight but rather improves regulation of glucose homeostasis. Hepatic deletion of miR-33 also increased circulating HDL-C levels which is consistent with the notion that ABCA1 is essential for RCT and is repressed by miR-33 [58].

Experiments showed that in certain lines of NSCLC (demonstrated in the A549 cell line), upregulated miR-106a targeted ABCA1 transporter, which induced cisplatin resistance in the cell lines [30]. ABCA1 had the lowest expression levels of all the evaluated ABC transporter genes in the investigated cells compared to controls (ABCA1, ABCC5, ABCC6, ABCC9, ABCD2, ABCG2 and ABCG4), showing that miR-106a specifically targeted *ABCA1* [30]. Overexpression of miR-106a significantly decreased *ABCA1* mRNA and protein expression in the evaluated cell lines, in line with the notion that miR-106a negatively regulates and targets ABCA1 [30].

MiR-200b-3p is another miRNA that directly targets ABCA1 transporter [6]. MiR-200b-3p expression was upregulated in tumor cells compared to adjacent normal tissues, facilitating lung adenocarcinoma cell proliferation and metastasis [6]. Therefore, miR-200b-3p should be evaluated as a novel molecular marker and therapeutic target for lung adenocarcinoma [6].

### 2.5. Low Density Lipoprotein Receptor-Related Proteins (LRPs)

Low density lipoprotein receptor-related proteins (LRPs) play an important role in the RCT mechanism. It was determined that *Lrp1* knockout in murine models is associated with reduced HDL secretion and decreased cell-surface localization of Abca1 without a change in total cellular Abca1 content [59]. LRPs have been explored and evaluated in various research projects due to their potential impact on development and progression of cancer. A family of LRPs have been found to play a role in cancer pathophysiology, including LRP1, LRP2, and LRP10 [60]. Several studies identified a new potential tumor suppressor gene associated with LRPs in NSCLC lines: lipoprotein receptor-related protein-deleted in tumors (LRP-DIP) [61]. Homozygous deletions of LRP-DIP were identified in 17% of NSCLC [61]. No LRP-DIP alterations were noted in SCLC.

A higher level of LRP1 protein may be associated with a higher endocytosis of upregulated transporter proteins at the cell surface, which is likely the cause of increased doxorubicin and emodin (an anti-inflammatory agent) accumulation and growth inhibition in lung adenocarcinoma and colorectal carcinoma cells [62]. Additionally, emodin was shown to upregulate LRP1 in a prostate cancer cell as well as nonprostate cell lines A549 (lung), HCT-15 (colon) and MG-63 (bone) under normoxic and hypoxia-like conditions via reactive oxygen species (ROS) generation [63]. Therefore, LRP1 expression may be a point for interventions to promote efficacy of anticancer drugs by allowing their more efficient uptake in lung adenocarcinoma and colorectal carcinoma [62,63].

Evaluation of *LRP1* mRNA levels showed a significant decrease in lung tumors compared to nontumorous lung tissue [64]. Additionally, a lower expression of LRP1 in lung adenocarcinomas correlates with less favorable clinical outcomes, and higher levels correlate to more favorable clinical outcomes [64].

However, evaluation of provisional databases in the Cancer Genome Atlas (TCGA) showed that in nine of ten cancers studied, a statistically significant correlation between *LRP1* mRNA expression and patient survival was observed only in bladder urothelial carcinomas [60]. It was reported that high levels of LDL receptor mRNA were associated with a decreased patient survival in pancreatic adenocarcinomas; high levels of *LRP10* mRNA were associated with a decreased patient survival in hepatocellular carcinomas, lung adenocarcinomas, and pancreatic adenocarcinomas [60]. LRP2 was the only LRP positively correlated to the improved patient survival in renal clear cell carcinomas. Therefore, these contradictions necessitate further evaluation of LRPs in general, and LRP1 specifically to determine how definite positive correlation with improved outcome is, and what variables can be changed to influence it.

## 3. Genes Underexplored for Their Roles in Lung Cancer Biology

### 3.1. Lysosomal Acid Lipase (LAL)

Lysosomal acid lipase (LAL) is a key enzyme in the metabolic pathway of neutral lipids, and is closely connected with regulation of homeostasis, immune response, and tumor progression in lung tissues [65]. Research further confirmed that *Lal*-deficient mice show an increase in tumor growth and metastasis associated with expansion of MDSCs [65]. Thus, LAL can behave like a tumor suppressor. Metabolic signaling in myeloid-derived suppressor cells (MDSCs) in *Lal* deficient mice was inhibited by PPAR-gamma ligand treatment [66]. This effect was mediated by regulating the mammalian target of rapamycin (mTOR) pathway, resulting in blocking MDSCs ROS overproduction [66]. Knockdown of mTOR in LAL deficient MDSCs suppressed their stimulation on proliferation of several cancer lineages, including B16 melanoma, Lewis lung carcinoma and transgenic mouse prostate cancer-C2 cancer cells [67]. Likewise, LAL deficiency promotes growth and metastasis in a melanoma model system [68]. Therefore, while LAL plays a role in pathophysiology of several types of cancers, its role in lung cancer biology needs to be further explored.

### 3.2. Niemann Pick Type C-1 (NPC1)

Niemann Pick Type C-1 (NPC1) plays a role in the efflux of cholesterol from lysosomes following LDL receptor-mediated endocytosis [69]. While NPC1 is a membrane-bound protein, and NPC2 is a soluble intracellular protein, both are necessary for the efflux of cholesterol from lysosomes [69]. Mutations in *NPC1* or *NPC2* classically result in an inherited lysosomal storage disease, Niemann-Pick disease type C [70].

While a strong connection of NPC1 to lung cancer has not been established at this time, research showed various connections to other cancers and to cancer treatments that could be further explored. The commonly used antifungal agent itraconazole has shown potential antitumor activity in the setting of inhibiting angiogenesis and lymphangiogenesis [71]. Itraconazole appears to act by directly targeting NPC1, along with VDAC1 which mediates mitochondrial ATP production, to shut off cholesterol trafficking motifs from the lysosome to the plasma membrane [71,72,73,74]. This combined activity subsequently leads to the downregulation of the mTOR pathway in endothelial cells, orchestrating the previously mentioned antitumor effects [71,72,73,74]. In other studies, itraconazole was found to counter tumor growth through perturbation of cholesterol trafficking in NSCLC endothelial cells, inhibiting migration, proliferation, and tube formation in response to growth factor stimulation [72]. We speculate that NPC1 likely mediates the itraconazole activity seen in NSCLC endothelial cells. In the same studies utilizing NSCLC endothelial cells, itraconazole has also appeared to enhance the antitumor efficacy of cisplatin [72].

Research showed a weak connection between esophageal adenocarcinoma and NPC1. In a cohort of 55 patients, two biopsies of esophageal adenocarcinomas exhibited a novel fusion gene as a result of a complex interchromosomal translocation of *NPC1* and maternal embryonic leucine zipper kinase (*MELK*) [75].

The approved anti-inflammatory and cancer management drug, cepharanthine, has been found to inhibit endolysosomal trafficking of LDL and free cholesterol via binding and inhibiting NPC1, subsequently increasing the lysosomal pH [76]. The resulting blockade caused a cholesterol-dependent dissociation of mTOR from the lysosomes, halting its downstream signaling, inhibiting angiogenesis and ultimately tumor growth [76]. Additionally, cepharanthine enhanced antitumor activity of cisplatin in murine models of breast and lung cancer [76].

### 3.3. StAR-Related Lipid Transfer Domain-3 (STARD3)

StAR-related lipid transfer domain-3 (STARD3) is a membrane-associated protein of late endosomes that creates endoplasmic reticulum-endosome contact sites [77,78]. Its activity causes cholesterol accumulation in endosomes for further trafficking elsewhere, removing it from the plasma membrane in the process [78]. It is suggested that STARD3 affects tumor progression via increasing cholesterol transport to mitochondria for steroidogenesis [78]. Beyond this however, it appears little is known about the potential mechanisms that link STARD3 with cancer progression.

While little information is available regarding the possible contribution of STARD3 to the pathophysiology of lung cancer, it has been associated with breast, colon, and gastric cancers. In the human genome, *STARD3* is less than 30 kilobases away from *HER2* on chromosome 17. Thus, it is perhaps not surprising that *STARD3* is classically co-amplified and co-expressed with *HER2* in approximately 10–25% of breast cancers [79,80]. It has been shown that inducing *STARD3* overexpression elevates *HER2* positive breast cancer proliferation, and ablating this expression returns an opposite effect [78,81]. In 21.3% of primary human gastric cancers, there exists a *PPP1R1B-STARD3* fusion gene, and its overexpression causes increased cell proliferation by way of PI3K/Akt activation [82]. The first STARD3 inhibitor to be analyzed, a compound named VS1, was shown to bind to STARD3 and steer it for degradation, causing antiproliferative activity in colon and breast cancer cell lines [83].

### 3.4. Sphingomyelin Phosphodiesterase 1 Gene (SMPD1)

Sphingomyelin is commonly found in animal cell membranes and has been shown to have many cellular functions, ranging from signal transduction to apoptosis [84]. It is under the regulation of the sphingomyelin phosphodiesterase 1 gene (*SMPD1*), which codes for an acid sphingomyelinase (ASM) that hydrolyzes sphingomyelin to phosphorylcholine and ceramide. Deficiency of ASM thus causes a lysosomal accumulation of sphingomyelin, and additionally a secondary accumulation of cholesterol [12].

Expression of *SMPD1* is correlated with a better overall survival rate in human lung cancer patients that received radiotherapy [85]. Further studies show that the miR-15a family is the likely causal agent of this increased expression, as miR-15a is negatively regulated by radiotherapy, and reciprocal elevations in SMPD1 result [85,86]. Induced inhibition of miR-15a has been shown to increase *SMPD1* expression both in vitro and in vivo within endothelial cells and enhance cell death [85,86].

ASM was found to be elevated in NSCLC cell lines, and downregulation of *SMPD1* conferred a resistance to serum-starvation mediated apoptosis [87]. A rodent model of lung adenocarcinoma showed that Asm inhibition reduced tumor development by way of Th1 and CD8+ enhancement [87].

Murine melanoma cell interactions with platelets have been shown to result in the secretion of Asm. The secreted Asm induces membrane structures saturated with ceramide to form on tumor cells. Such structures would cluster integrins and facilitate metastatic spread into the lung [88]. Transplantation of melanoma cells into wild-type mice showed multiple lung metastases, while *Smpd*1 knockout mice were protected under the same conditions [88]. Transplanting wild-type platelets and melanoma cells into *Smpd*1 knockout mice reinvigorated melanoma metastasis [88]. Further, it was shown that inhibition of ASM pharmacologically with amitriptyline prevented tumor metastasis [88].

Cisplatin, a front-line therapy of lung cancer, causes an ASM-dependent ceramide generation that induces apoptosis in HT29 (human colorectal adenocarcinoma) cells [89]. There is a dramatic increase in membrane fluidity following cisplatin treatment, and when subsequently inhibited by membrane stabilizing agents such as cholesterol, there is a prevention of Fas-mediated apoptosis [89]. Further detailed studies have shown that cisplatin-induced procaspase 8 cleavage is ASM dependent in NSCLC [90]. This is of important note as many NSCLC cell lines have altered expression of pro-apoptotic BAX and BAK, conferring a resistance to cisplatin. Thus, BAX and BAK expression might not be necessary in cisplatin-resistant NSCLC, as generation of ceramide can potentially induce apoptosis via caspase-8 activity [90].

5-FU resistant colorectal cancer cells were found to be associated with elevated levels of sphingomyelin and decreased levels of ceramide [91], and down-regulation of *SMPD1* was associated with resistance to treatment regimens that included 5-FU [91]. Similar direction of inquiry regarding primary lung cancer could be explored.

### 3.5. Neutral Cholesterol Ester Transferase 1 (NCEH1)

Neutral cholesterol ester transferase 1 (NCEH1) is a single-membrane-spanning membrane protein that plays an initial role in converting cholesterol esters (storage form) to free cholesterol (metabolic form) [92]. An elevated NCEH1 activity increases hydrolysis of cholesterol esters, and knockdown of NCEH1 logically induces elevated cholesterol ester levels [93,94]. Expression of NCEH1 is ubiquitous throughout the human body [95]. While a strong connection between NCEH1 and lung cancer has not been established yet, its overexpression has been linked to many cancer types including ovarian [96] and breast [97] cancers.

KIAA1363 is a serine hydrolase enzyme containing an ether lipid signaling motif and has been shown to be elevated in aggressive ovarian and melanoma cell lines [98]. Investigation shows that KIAA1363 is likely linked to NCEH1 pathways, as KIAA1363 inhibition results in prostate tumor suppression activity in vivo, likely through downregulation of monoalkylglycerol ether classes of neutral ether lipids [99].

How NCEH1 functions in cancer growth is unclear; however, it has been shown that NCEH1 deletions upregulate levels of cholesterol esters, which play a role in signaling ER stress and apoptosis in macrophages [93]. Aberrant homeostasis of lipid levels in macrophages classically results in the formation of foam cells and subsequent atherosclerosis [100].

NCEH1 (along with RARRES3, NTN4, CFB, and CYP4ZJ) are frequently co-expressed with TNF superfamily member 10 (TNFSF10 also known as TRAIL) in breast cancer patients [101]. TNFSF10 is involved in p53-dependent apoptosis through its identity as a transcriptional target gene of p53; studies show upregulation of TNFSF10 and subsequent p53-mediated apoptosis upon 5-FU and adriamycin treatment [102]. MiR-7641 transfection downregulated both TNFSF10 and NCEH1 expression, in line with the idea of a common mechanism that regulates the expression of both NCEH1 and TNFSF10 [102].

### 3.6. Scavenger Receptor Class B Type I (SR-BI)

Scavenger receptor class B type I (SR-BI), an integral membrane glycoprotein receptor, plays an essential role in reverse cholesterol transport. It is understood that SR-BI binds HDL molecules to remove cholesterol esters via the formation of a non-aqueous channel for transport into the liver [103]. Additionally, SR-BI has been shown to facilitate the selective uptake of cholesterol by malignant cells, including breast, prostate, and pancreatic cancer [103]. Observational studies have shown that higher SR-BI expression levels are associated with more aggressive lung adenocarcinomas with poorer prognoses [104]. There is mounting support and evidence for the idea that cancer cells require increased cholesterol supplies, alongside specific changes in lipid and cholesterol metabolism [105].

The mechanism of SR-BI-mediated transport of HDL cholesterol esters makes way for therapeutics to overcome faulty endo-lysosomal uptake routes, such as what was done by Lacko et al. whom engineered a paclitaxel-loaded reconstituted HDL vessel that was taken up by SR-BI expressing prostate cancer cell lines [106]. A similar approach was taken by Zhang et al. who successfully used dichloroacetate in a dual-targeting system of a reconstituted HDL that could also recognize SR-BI to successfully bring an antitumor payload to human lung adenocarcinoma in a murine model [107]. One study was able to inhibit human lung cancer cell growth through a combination of SR-BI overexpression and anti-neoplastic alpha-Tocopheryl-succinate inoculation in A549 cells both in vitro and in vivo [108]. However, it is unfortunate to note that the success rate of these drug types completing clinical trials successfully is substantially low, due to the off-target effects that these drugs often have against normal tissue [109].

SR-BI has been shown to be involved in some subtypes of breast cancer, wherein its increased expression resulted in elevated cholesterol esterification alongside elevated expression of the LDL receptor [110]. Other studies have shown that decreased plasma cholesterol levels, specifically lower HDL cholesterol levels, have been found in a plethora of cancer types, including lung cancer [105]. Causes of this finding of lowered HDL cholesterol levels in cancer patients have been attributed at least partially to overexpression of SR-B1 [110,111,112]. Further, it has been shown in human breast cancer cell lines that elevated HDL levels are associated with an increased risk of breast cancer development [113]. This raises the potential question of whether elevated SR-BI expression is a result of a need for increased cholesterol uptake from HDL, thus feeding further tumor growth [113].

### 3.7. Cholesterol Efflux Factors and Targeted and Immunotherapy of Lung Cancer

The discussion so far has been primarily focused on chemotherapy and radiotherapy. The literature is sparse on direct connections between cholesterol efflux factors and targeted or immunotherapy of lung cancer. However, agonism of the cholesterol efflux regulator LxR has been shown to sensitize lung cancer cells to epidermal growth factor receptor (EGFR) tyrosine kinase inhibitor (TKI) treatment [114,115]. More intriguingly, therapeutic LxR agonism reduced myeloid-derived suppressor cells (MDSC) abundance in murine models and in patients treated in a first-in-human dose escalation phase 1 trial [116]. Considering that MDSCs are an immunosuppressive innate cell population and that immunotherapy non-responders often harbor high levels of circulating MDSCs, the LxR/ApoE axis is implicated in the regulation of innate immune suppression and as a target for enhancing the efficacy of cancer immunotherapy in patients [116].

## 4. Conclusions

**Figure 1:**
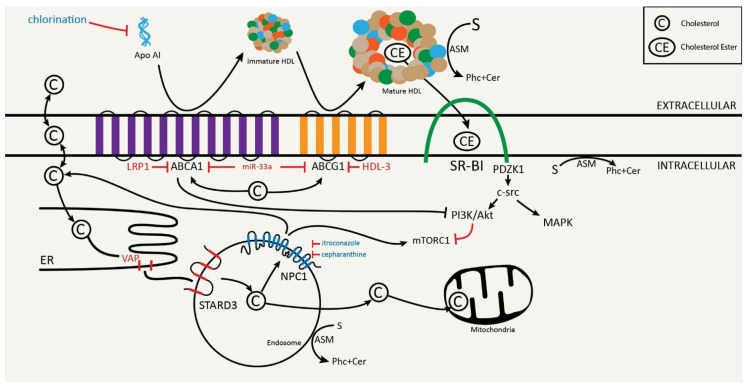
The interplay of common cholesterol efflux factors and their relationship with selected chemotherapeutic agents. Cholesterol (C) can be effluxed from the cell via a variety of mechanisms including passive diffusion, SR-B1-facilitated diffusion, ABCA1 active reflux, and ABCG1-mediated efflux. Apo AI together with ABCA1 provides the rate-limiting step of RCT, with ABCG1 assisting in HDL lipidation. Both ABCA1 and ABCG1 are regulated by miR-33a. SR-B1 is able to bind HDL molecules and remove cholesterol esters from the HDL molecule for intracellular transport. Downstream effects of SR-B1 activity shows enhanced cellular proliferation and migration through MAPK and PI3K/Akt pathways, with the latter being shown to downregulate the mTORC1 pathway. Cholesterol movement involving cellular endosomes has been shown to be regulated by STARD3 and NPC1. STARD3 complexes with VAP on the ER to allow for cholesterol movement from the ER into the endosome, and has been shown to further move this cholesterol into the mitochondria for steroidogenesis. NPC1 mediates cholesterol efflux from the endosome, and has been shown to upregulate the mTORC1 pathway in the process. ASM, coded for by the SMPD1 gene, is found on HDL molecules, the plasma membrane, and cellular endosomes. ASM serves to convert sphingomyelin (S), formed in part by cholesterol, into phosphorylcholine (Phc) and ceramide (Cer).

**Figure 2:**
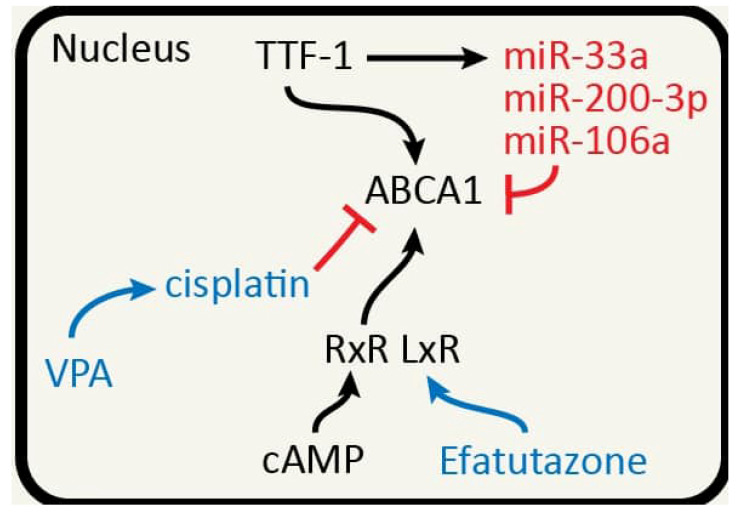
The connection between TTF-1 and ABCA1. ABCA1 has been shown to be negatively regulated through miRNAs including miR-33a, miR-200-3p, and miR-106a. Lai et al. suggested that TTF-1, miR-33a, and ABCA1 may form an incoherent feed-forward loop (TTF-1 → ABCA1; TTF-1 → miR-33a—|ABCA1) [22]. Treatment with Valproic Acid (VPA) enhanced cisplatin sensitivity of non-small-cell lung cancer (NSCLC) cells via HDAC2 mediated down-regulation of ABCA1. Efatutazone treatment increased mRNA and protein expression of PPAR-gamma, LxR-alpha and ABCA1, suggesting that Efatutazone functions through PPAR-gamma/LxR-alpha/ABCA1 pathway. cAMP also can regulate ABCA1 expression. The fact that TTF-1 is a lung lineage master regulator highlights the fact that cholesterol efflux factors may be regulated in a tissue-specific manner.

The fact that the monogenic Tangier disease is attributed to mutations in the *ABCA1* gene proves that ABCA1-mediated cholesterol efflux is an essential link in balancing the physiological well-being of humans (see Figure 1 and Figure 2 for illustrations of the main concepts discussed in this article). Moreover, it is well established that cholesterol efflux is relied upon to minimize the potential harm of excess free cholesterol to individual cells. Cancer cells have evolved to exploit this essential link to facilitate multiple phenotypes associated with malignancies. We understand that there are other important molecules (e.g., LxR) that regulate cholesterol efflux factors. We chose to not delve into these molecules in this article because they have been extensively reviewed elsewhere [117,118]. With regard to one of the most common solid tumors, i.e., lung cancer, we are only beginning to appreciate the roles of cholesterol efflux in lung tumorigenesis and treatment strategies/responses. It is our prediction that future research on the interplays between cholesterol efflux and lung cancer will expose novel entry points for drug discovery and treatment optimization.

## Figures and Tables

**Table 1:** Major cholesterol efflux genes and their existing connection to lung cancer biology.

Gene Symbol	Protein	Established Connection to Lung Cancer Biology in Literature
ABCA1	ATP binding cassette A1	Strong
Apo AI	Apolipoprotein AI	Strong
ABCG1	ATP binding cassette G1	Strong
miRNA-33	Micro RNA-33	Strong
LRP1	LDL receptor related protein 1	Strong
LIPA	Lysosomal acid lipase A	Weak
NPC1	Niemann Pick Type C-1	Weak
STARD3	Steroidogenic acute regulatory D3	Weak
SMPD1	Sphingomyelinase phosphodiesterase 1	Weak
NCEH1	Neutral cholesteryl ester hydrolase	Weak
SR-BI	Scavenger Receptor BI	Weak
